# Inhibition of Death-associated Protein Kinase 1 protects against Epileptic Seizures in mice

**DOI:** 10.7150/ijbs.59922

**Published:** 2021-06-11

**Authors:** Chen-Ling Gan, Yulian Zou, Yongfang Xia, Tao Zhang, Dongmei Chen, Guihua Lan, Yingxue Mei, Long Wang, Xindong Shui, Li Hu, Hekun Liu, Tae Ho Lee

**Affiliations:** 1 Fujian Key Laboratory for Translational Research in Cancer and Neurodegenerative Diseases, Institute for Translational Medicine, School of Basic Medical Sciences, Fujian Medical University, Fuzhou, Fujian 350122, China.; 2 Fujian Provincial Key Laboratory of Natural Medicine Pharmacology, Institute of Materia Medical, School of Pharmacy, Fujian Medical University, Fuzhou, Fujian 350122, China.; 3 Immunotherapy Institute, Fujian Medical University, Fuzhou, Fujian 350122, China.

**Keywords:** Death-associated protein kinase 1, epilepsy, kindling, pentylenetetrazol, phosphorylation, seizure

## Abstract

Epilepsy is a chronic encephalopathy and one of the most common neurological disorders. Death-associated protein kinase 1 (DAPK1) expression has been shown to be upregulated in the brains of human epilepsy patients compared with those of normal subjects. However, little is known about the impact of DAPK1 on epileptic seizure conditions. In this study, we aim to clarify whether and how DAPK1 is regulated in epilepsy and whether targeting DAPK1 expression or activity has a protective effect against epilepsy using seizure animal models. Here, we found that cortical and hippocampal DAPK1 activity but not DAPK1 expression was increased immediately after convulsive pentylenetetrazol (PTZ) exposure in mice. However, DAPK1 overexpression was found after chronic low-dose PTZ insults during the kindling paradigm. The suppression of DAPK1 expression by genetic knockout significantly reduced PTZ-induced seizure phenotypes and the development of kindled seizures. Moreover, pharmacological inhibition of DAPK1 activity exerted rapid antiepileptic effects in both acute and chronic epilepsy mouse models. Mechanistically, PTZ stimulated the phosphorylation of NR2B through DAPK1 activation. Combined together, these results suggest that DAPK1 regulation is a novel mechanism for the control of both acute and chronic epilepsy and provide new therapeutic strategies for the treatment of human epilepsy.

## Introduction

Epilepsy is a chronic, repetitive and sudden brain dysfunction characterized by International Antiepileptic Alliance guidelines as recurrent unprovoked seizures 1. Approximately 1% of the global population is affected by this disease, which is second to stroke among common neurological diseases 2. Epilepsy seriously impairs the physical and mental health of patients, thereby affecting their quality of life, and places a heavy burden on families, society and government 3, 4. One-third of epilepsy patients who are resistant to multiple traditional antiepileptic drugs gradually develop drug-resistant epilepsy, which is also known as refractory epilepsy 5, 6. Once refractory epilepsy develops, treatment options become extremely limited. Although surgical removal of the lesion is an effective method for the treatment of refractory epilepsy, it is only applicable to some patients and postoperative recurrence and irreversible brain damage may occur 7-9.

Most traditional first-line antiepileptic drugs show severe side effects and drug resistance 10, 11. Carbamazepine (CBZ) has been used to treat grand mal seizures and psychomotor epilepsy 12. Long-term application of CBZ is associated with a risk of developing aplastic anemia 13. Valproic acid is an effective antiepileptic drug mainly used for generalized epilepsies, but shows dose-dependent teratogenic effects and a rare, but fatal hepatotoxicity 14, 15. Phenytoin, a barbiturate derivative used in treatment of status epilepticus, shows clinical side effects such as gingival hyperplasia 16, 17. Therefore, it is necessary to study antiepileptic drugs based on a new mechanism.

Death-associated protein kinase 1 (DAPK1) is a calcium/calmodulin-dependent serine/threonine kinase 18, 19. DAPK1 might have a critical role in neurogenesis, neuronal function and cell death 20, 21. Interestingly, inhibition of DAPK1 activity or expression attenuates neuronal cell death in a stroke mouse model and AD-related neuropathology in AD animal models, suggesting that inhibiting DAPK1 might protect neurons from neuronal damage 22-28. DAPK1 was first discovered as a novel neuronal death regulator in epilepsy, and subsequent clinical data have shown that DAPK1 expression levels are upregulated in epileptic individuals compared with age-matched normal subjects, indicating that DAPK1 may be closely involved in the pathophysiology of epilepsy 29, 30. Reports have indicated that DAPK1 interacts with tumor necrosis factor receptor 1 (TNFR1) or p53 and increases neuronal apoptosis in seizures 31, 32. Nevertheless, whether the expression or activity of DAPK1 is regulated in epilepsy remains incompletely understood and whether inhibition of the function of DAPK1 exerts antiepileptic effects in animal models has not been determined.

In the present study, we report for the first time that DAPK1 activity and expression are separately regulated depending on seizure conditions. Moreover, the DAPK1-NR2B axis plays a crucial role in the regulation of seizures in pentylenetetrazol (PTZ)-induced animal models. Consequently, DAPK1 ablation or treatment with a pharmacological DAPK1 activity inhibitor in mice dramatically reduces the development of seizures induced by PTZ. Taken together, our study demonstrates a novel role of DAPK1 regulation and offers a novel therapeutic strategy for treating human epilepsy by targeting DAPK1.

## Methods

### Materials

PTZ and DAPK1 inhibitor (4Z)-4-(3-pyridylmethylene)-2-styryl-oxazol-5-one were purchased from MilliporeSigma (St. Louis, MO, USA). The antibodies used in the current study are detailed in Supplementary Table 1.

### Phosphorylated Ser308-specific DAPK1 antibodies

Phosphorylated Ser308-specific DAPK1 antibodies were raised by immunizing mice with a KLH-coupled pSer308-containing DAPK1 peptide ARKKWKQSVRL (Abmart, Shanghai, China) and were affinity purified, as described 33.

### Animals

All experiments were carried out using wild-type (WT) and DAPK1 knockout (KO) mice on a C57BL/6 background. The mice were housed on a 12-hour light/dark schedule (7 am/7 pm) with food and water. The use of experimental animals was conducted according to the regulations of the Association for Assessment and Accreditation of Laboratory Animal Care International and approved by the Experimental Animal Ethics Committee of Fujian Medical University.

### Surgery and electrode implantation

Animals were anesthetized using isoflurane (1%) and fixed in a stereotaxic apparatus. Epidural electroencephalogram (EEG) electrodes were fixed in the following position relative to bregma: +2.0 mm anteroposterior (AP) and +1.5 mm mediolateral (ML). A reference electrode was fixed with dental cement at the right side in the following positions: -2.0 mm AP and +3.0 ML 34.

### PTZ dosing paradigms

PTZ was dissolved in 0.9% saline, filter sterilized and injected intraperitoneally (i.p.). Acute paradigm: mice were treated with a convulsive dose of PTZ (50 mg/kg) and monitored in Plexiglas cages for the next 15 min. Seizure severity was graded by a double-blind study to the experimental condition by using an improved scoring system. Stage 0: normal behavior; stage 1: immobility; stage 2: facial and neck jerks; stage 3: tail extension; stage 4: chronic seizure (sitting); stage 5: generalized chronic, tonic-clonic seizure (lying on belly); stage 6: generalized chronic, tonic-clonic seizure (lying on side); stage 7: full tonic extension; and stage 8: death 35-37. Kindling paradigm: mice were treated with a subconvulsive dose of PTZ (35 mg/kg) every other day, observed for the next 15 min and scored for seizure severity after each PTZ injection based on a modified Racine scale 38-40. Stage 0: normal behavior; stage 1: immobilization or facial myoclonus; stage 2: head nodding; stage 3: forelimb clonus or short myoclonic jerks; stage 4: rearing generalized chronic seizure; stage 5: generalized tonic-clonic seizures and falling with loss of posture; and stage 6: death. Mice that exhibited at least three consecutive convulsive seizures (≥ stage 4) within 17 days were considered fully kindled and assigned to the epilepsy group. The latency onset of the first stage 4 or 5 seizure, as well as the mean seizure stage, was calculated during the experimental course. Some mice were sacrificed 30 min after the fourth PTZ injection, while the rest of the mice continued to undergo behavioral testing until the end of the experiment (total 9 injections).

### EEG trace and behavioral analysis of seizures

EEG activity was continuously traced using an EEG recording system (Chengdu TME Technology, Chengdu, Sichuan, China) at a sampling rate of 5 kHz using an amplifier for 15 min after a convulsive dose of PTZ treatment as previous reported 41-44. EEGs signals were filtered at 10 to 300 Hz and analyzed using built-in analysis software (BL-420F Data Acquisition & Analysis System). EEG segments with continuous high-frequency and high-amplitude polyspikes lasting for at least 10 seconds were considered generalized seizures (stage 5-6) followed by postictal depression of the EEG 45, 46.

### Tissue preparation and immunohistochemical analysis

Mouse brains were harvested and postfixed in 4% paraformaldehyde overnight. Tissue sections were embedded in paraffin after immersion fixation. After deparaffinizing and rehydrating sections by xylene and descending grades of ethanol, they were quenched endogenous peroxidase activity by with 3% H_2_O_2_/PBS. Tissue sections were incubated in 5% horse serum, 5% bovine serum albumin (BSA) and 0.1% v/v Tween 20 in PBS after antigen retrieval. Primary antibody was incubated with sections in blocking buffer overnight at 4 °C and then the secondary antibody was biotinylated goat-anti-mouse or rabbit IgG. Sections were treated with ABC reagents and the horseradish peroxidase (HRP) (Vector Laboratories, Burlingame, CA, USA). Positively stained areas were quantified with Image-Pro Plus.

### Immunoblot analysis

Mouse brains were lysed in ice-cold buffer (10 mM Tris-HCl (pH 7.4), 0.8 M NaCl, 1 mM EGTA, 10% sucrose, and 1 mM DTT) supplemented with protease inhibitor and phosphatase inhibitor cocktails. Protein samples (5-10 μg) were separated by SDS-PAGE and transferred to 0.45 μm polyvinylidene fluoride (PVDF) membranes (MilliporeSigma). The membranes were incubated with various primary antibodies and subsequently HRP-conjugated secondary antibodies. Proteins were detected using Immobilon Western HRP ECL solution (MilliporeSigma) on ChemiDoc XRS+ (Bio-Rad, Hercules, CA, USA). Protein levels were quantified by densitometry using Fiji/ImageJ Coloc 2.

### Immunoprecipitation analysis

Mouse brain lysates were added to the corresponding antibodies and incubated for 3 h at 4 °C with gentle rotation followed by protein A/G-Sepharose mixture for 1 h. The beads were washed three times, and the collected immune complexes were then resolved by using SDS-PAGE for the immunoblot analysis.

### Statistical analysis

All experimental data were input into GraphPad Prism 8 software for statistical analysis. Statistical evaluation was performed using two-tailed Student's t test or one-way ANOVA with Tukey's multiple comparisons test. All data are presented as the mean ± standard error, and statistical significance was identified at p < 0.05.

## Results

### DAPK1 activity is increased by acute single PTZ administration

**Figure 1:**
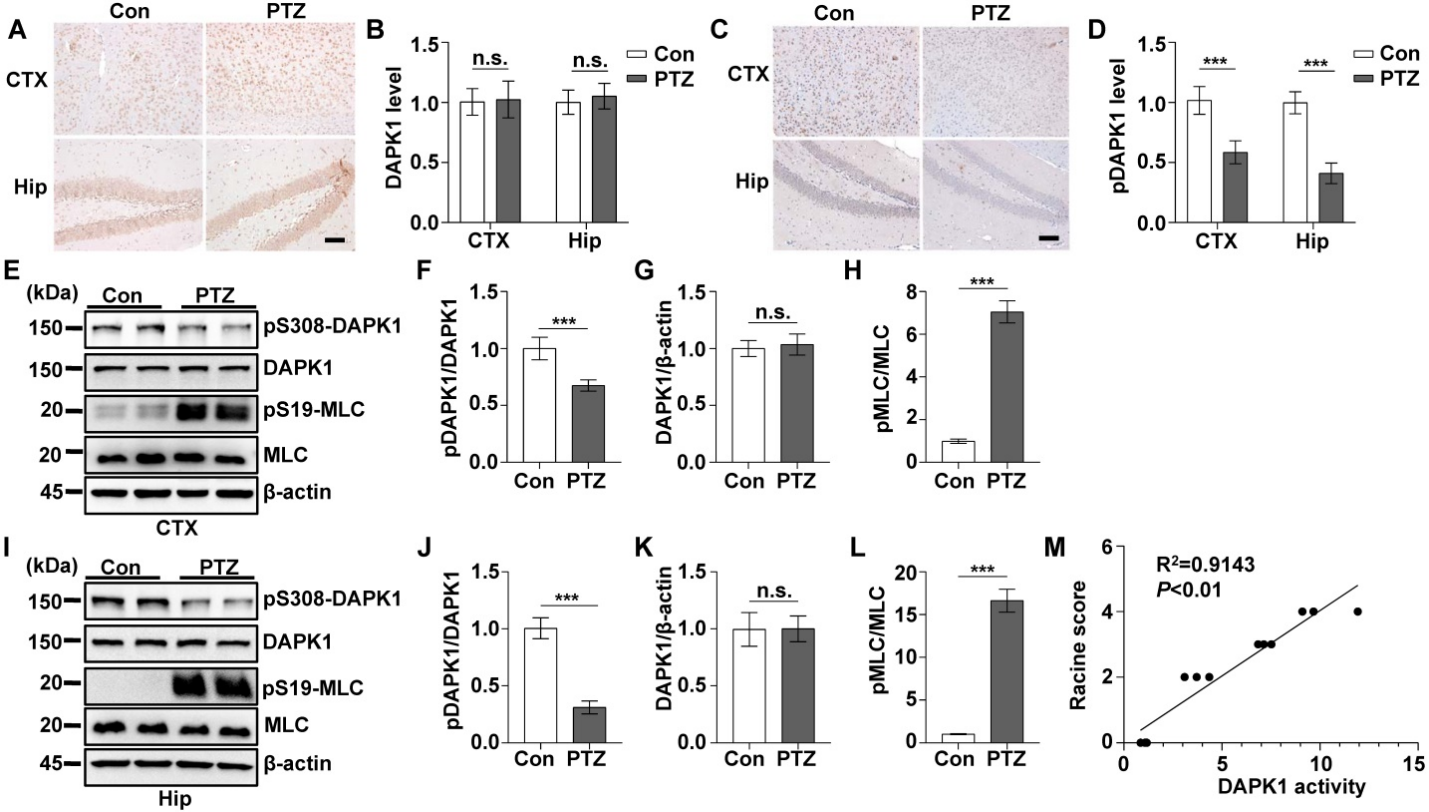
** Acute PTZ administration increases DAPK1 activity.** Mice (C57BL/6, male, P56-P80, n = 8/group) were administered a convulsive dose of PTZ (50 mg/kg), and brain tissues were harvested 3 min after PTZ injection. **A-D** Immunohistochemistry using an anti-DAPK1 (**A**) and anti-pSer308-DAPK1 (**C**) antibodies was conducted on paraffin-embedded cortical and hippocampal sections from the control and PTZ-treated mice. Scale Bar = 100 µm. Quantitation of DAPK1 **(B)** and pSer308-DAPK1 (**D**) staining intensity (arbitrary units; two-tailed Student's t-tests). **E-L** Cortical and hippocampal lysates were subjected to immunoblotting analysis with anti-pSer308-DAPK1, anti-DAPK1, anti-pSer19-MLC, anti-MLC or anti-β-actin antibodies (^***^P < 0.001 *vs* control; two-tailed Student's t-tests). **M** Correlation between the Racine score on the Y axis and DAPK1 activity on the X axis based on the pSer19-MLC level (R^2^ = 0.9143, *P* < 0.01, Pearson correlation coefficient). n.s., no significance. All data shown represent the means ± standard errors of three independent experiments.

According to the EEG and behavioral characteristics, a few minutes are required for mice to develop from a normal state to a generalized tonic-clonic seizure (GTCS), which is followed by postictal generalized EEG suppression (PGES) under the action of PTZ 37. In this very short period of time, mice undergo two kinds of drastically different EEG and behavioral changes and corresponding pathological changes: GTCS with neuronal hyperexcitability and PGES. Because the expression of DAPK1 is increased in epilepsy patients, we first characterized the short-term changes in DAPK1 expression after acute PTZ treatment. A convulsive dose of PTZ (50 mg/kg) was injected into WT mice, and DAPK1 protein levels were measured in the brain using immunohistochemistry and immunoblotting assays. Surprisingly, the administration of PTZ did not change DAPK1 protein expression in the cortex or hippocampus (Fig. 1A and B). The immunoblotting results also confirmed that DAPK1 levels were not different between untreated and PTZ-injected mice in the brain (Fig. 1E, G, I, and K). Reports have indicated that the activity of DAPK1 is regulated by posttranslational phosphorylation 19, 23, 24. In particular, the autophosphorylation of DAPK1 at Ser308 negatively regulates DAPK1 activity. Dephosphorylation at Ser308 in DAPK1 enhances the phosphorylation of myosin light chain (MLC), which is a substrate of DAPK1 and generally considered to be a characteristic of the catalytic activity of DAPK1 19, 23, 24. We further examined DAPK1 activity after PTZ administration in the mouse brain by immunohistochemistry and immunoblotting assays and found that PTZ insult dramatically decreased phosphorylated Ser308 levels in both the cortex and hippocampus compared to that in the control mouse brain (Fig. 1C-F, I, and J). Moreover, phosphorylated MLC levels were significantly upregulated in PTZ-injected mice (Fig. 1E, H, I, and L). Furthermore, positive correlations occurred between DAPK1 activity and seizure grade induced by a single injection of PTZ as revealed by the Pearson correlation coefficient (R^2^ = 0.9143) (Fig. 1M). Taken together, our data show that acute PTZ administration induces the catalytic activity of DAPK1 but not the expression of DAPK1 in mouse brains.

### DAPK1 ablation in mice exhibits decreased susceptibility to seizures induced by PTZ

**Figure 2:**
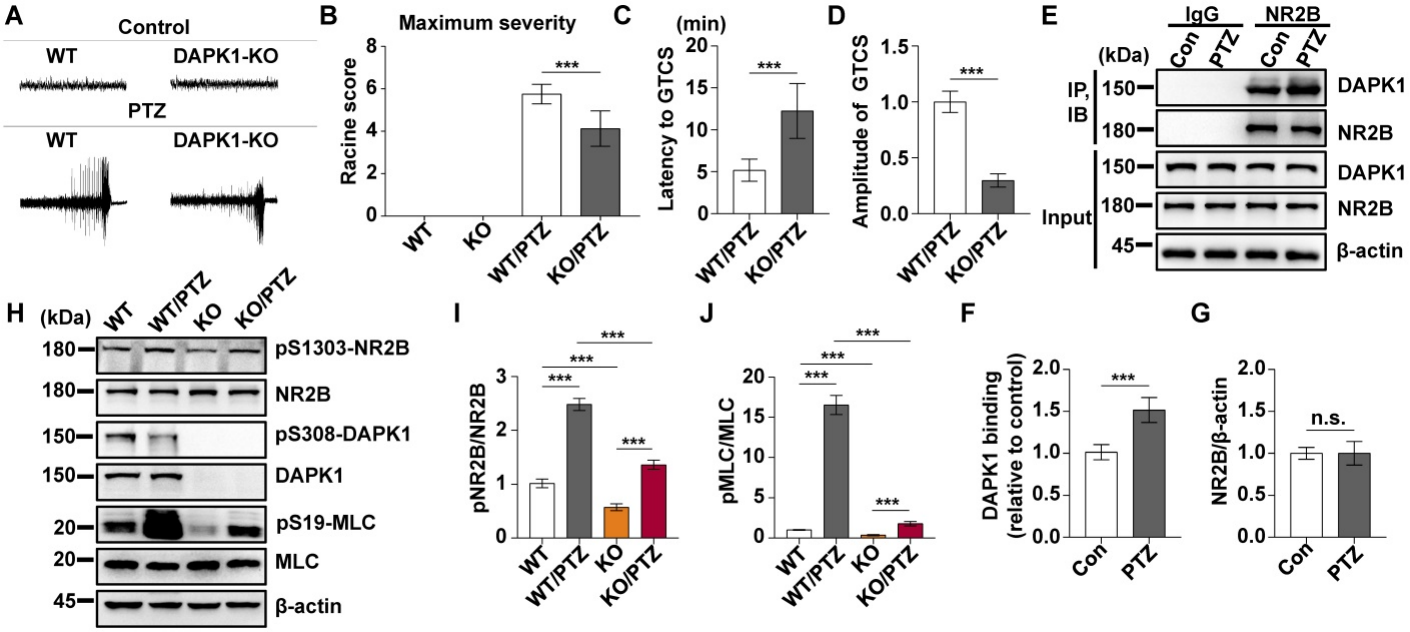
** DAPK1 deletion exerts rapid anti-seizure effects in mice.** Mice (C57BL/6, male, P56-P80, n = 12/group) were implanted with electrodes, allowed to recover for 7 days, and then insulted by a convulsive dose of PTZ (50 mg/kg). Brain tissues from a number of the animals (n = 4) were collected 3 min after PTZ administration for immunoblotting and immunoprecipitation analysis, and the remaining animals continued to undergo behavioral analysis and EEG recording for 15 min. **A** Representative EEG traces from saline or PTZ administration are shown for WT and DAPK1 KO mice. **B-D** DAPK1 deletion mice displayed lower seizure scores (**B**), longer latency to GTCS (**C**) and lower amplitude of GTCS post PTZ administration compared to WT littermates (**D**) (^***^P < 0.001 *vs* control, two-tailed Student's t-tests). **E-G** Enhanced interaction between NR2B and DAPK1 in the hippocampus after PTZ exposure (^***^P < 0.001 *vs* control, two-tailed Student's t tests). **H-J** Brain lysates were subjected to immunoblotting analysis with anti-pSer1303-NR2B, anti-NR2B, anti-pSer308-DAPK1, anti-DAPK1, anti-pSer19-MLC, anti-MLC or anti-β-actin antibodies. Statistical significance was determined by one-way ANOVA with Tukey's multiple comparisons tests (^***^P < 0.001). n.s., no significance. All data shown represent the means ± standard errors of three independent experiments.

We showed that a convulsive dose of PTZ injection increased the activity of DAPK1 in the mouse brain (Fig. 1). To investigate whether DAPK1 regulates seizures after PTZ administration, we examined the effects of DAPK1 KO on PTZ-related seizure phenotypes in mice. Mice exposed to PTZ insult showed a series of behaviors with seizure characteristics, beginning with whisker trembling and sudden behavioral arrest, which eventually progressed to GTCS. More than 85% of the WT mice displayed a single generalized motor seizure recorded by behavior observation and on EEG (Fig. 2A). However, DAPK1 ablation resulted in lower seizure scores (mean seizure grade 4.2) and a significantly longer latency to GTCS (mean latency 12.2 min) than WT littermates (Fig. 2B and C). EEGs indicated that DAPK1 KO mice showed a lower amplitude of electrographic seizures (Fig. 2D), suggesting that DAPK1 gene KO has a protective role against a convulsive dose of PTZ. Given that DAPK1 KO attenuates PTZ-induced seizure phenotypes, a critical question is how DAPK1 regulates seizures after PTZ. Growing evidence suggests that upregulation of the NR2B subunit of the N-methyl-D aspartate receptor (NMDAR) accounts for enhanced seizure susceptibility 47-49. Reports have indicated that DAPK1 binds to and phosphorylates NR2B and induces brain damage by ischemic stroke 22. To elucidate the molecular mechanisms how DAPK1 controls PTZ-mediated seizures, we examined the NR2B phosphorylation. Compared with that in the control mice, there was an enhanced interplay between DAPK1 and NR2B in the hippocampus after PTZ (Fig. 2E-G). Interestingly, the phosphorylation of NR2B at Ser1303 was significantly increased after PTZ in WT mice but not in DAPK1 KO mice (Fig. 2H and I). The levels of phosphorylated MLC in the WT mice were increased after PTZ compared to that in the saline-treated mice, while DAPK1 deletion mice displayed decreased phosphorylated MLC levels similar to that of the WT mice (Fig. 2H and J). Thus, the above results indicate that PTZ stimulates the phosphorylation of NR2B through DAPK1 and that the suppression of DAPK1 expression may confer protective effects against the seizure caused by PTZ.

### Pharmacological inhibition of DAPK1 activity exerts rapid anti-seizure effects

**Figure 3:**
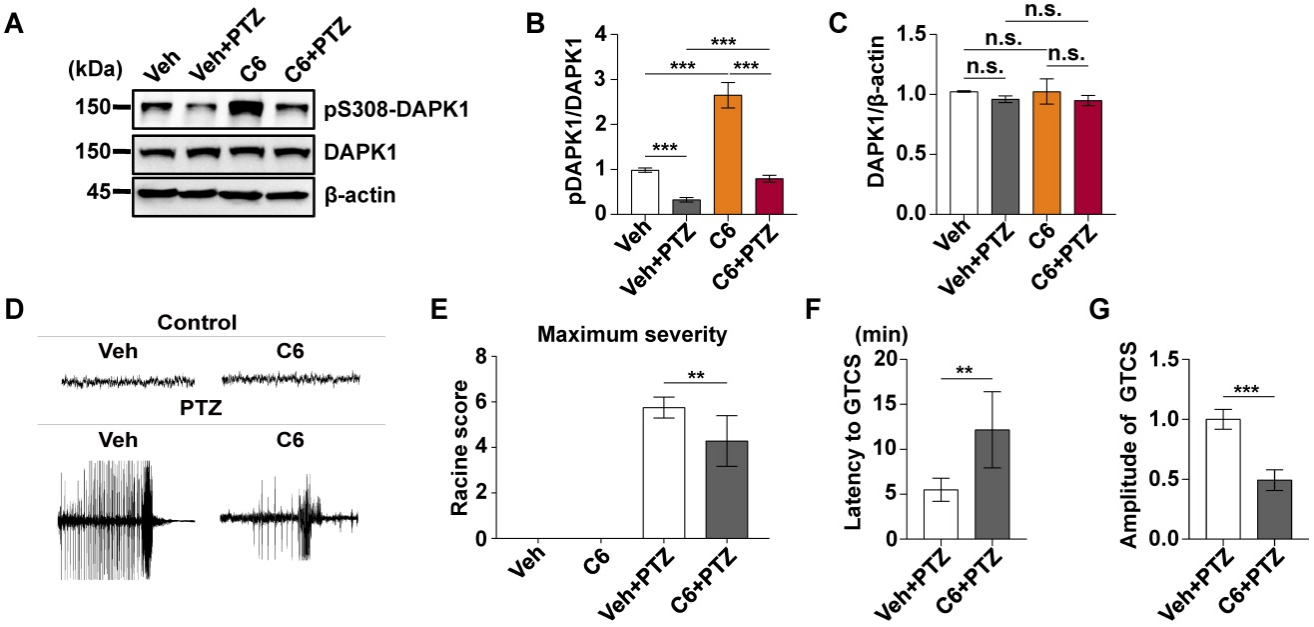
** DAPK1 inhibition exerts rapid anti-seizure effects.** Mice (C57BL/6, male, P56-P80, n = 12/group) were implanted with electrodes, allowed to recover for 7 days, and then pretreated with a DAPK1 inhibitor, vehicle or C6 (10 mg/kg) for 30 min followed by a convulsive dose of PTZ (50 mg/kg). Brain tissues from a number of the animals (n = 4) were collected 3 min after PTZ administration for immunoblotting analysis, and the remaining animals continued to undergo behavioral analysis and EEG recording for 15 min. **A-C** C6 pretreatment decreases DAPK1 activity after PTZ. The brain lysates were subjected to immunoblotting analysis with anti-pSer308-DAPK1, anti-DAPK1, or anti-β-actin antibodies. Statistical significance was determined by one-way ANOVA with Tukey's multiple comparisons tests (^***^P < 0.001). **D** Representative EEG traces from saline or PTZ are shown for the vehicle- or DAPK1 inhibitor-treated group. **E-G** DAPK1 inhibitor-treated mice displayed lower seizure scores (**E**), longer latency to GTCS (**F**) and lower amplitude of GTCS (**G**) post PTZ administration. Statistical significance was determined by one-way ANOVA with Tukey's multiple comparisons test (**E**) or two-tailed Student's t tests (**F** and **G**) (^**^P < 0.01 and ^***^P < 0.001). n.s., no significance. All data shown represent the means ± standard errors of three independent experiments.

Since DAPK1 KO dramatically attenuates PTZ-induced seizure pathology in mice, we next investigated whether the small molecule DAPK1 kinase inhibitor ameliorates seizure effects in animal models. The DAPK1 inhibitor (4*Z*)-4-(3-pyridylmethylene)-2-styryl-oxazol-5-one (C6) was chosen because it effectively inhibits DAPK1 catalytic activity 50. We previously showed that this compound selectively suppresses DAPK1 function and inhibits DAPK1-mediated tau phosphorylation 51. C6 was intraperitoneally injected into WT mice at a dose of 10 mg/kg for 30 min before PTZ treatment (Supplementary Fig. 1). Compared with the untreated mice, the DAPK1 inhibitor increased the levels of phosphorylated DAPK1 at Ser308, which negatively regulates DAPK1 activity in the mouse hippocampal brain (Fig. 3A-C). Interestingly, the phosphorylation of DAPK1 at Ser308 was effectively increased, even after PTZ treatment with the DAPK1 inhibitor (Fig. 3A-C). The anticonvulsant effect of the DAPK1 inhibitor could significantly reverse the formation of GTCS without affecting EEG under normal conditions; typical characteristics of generalized motor seizures were not observed in approximately half of the animals under DAPK1 inhibitor pretreatment; and only intermittently increased spider-wave discharge was recorded on EEG (Fig. 3D). Moreover, C6 pretreatment decreased the seizure grade (mean seizure grade of 4.3) induced by PTZ insult (Fig. 3E-G). The results demonstrate that suppressing DAPK1 activity may have potential as a therapeutic option for reducing susceptibility to epilepsy.

### Repeated PTZ treatment increases DAPK1 protein expression in the mouse brain

**Figure 4:**
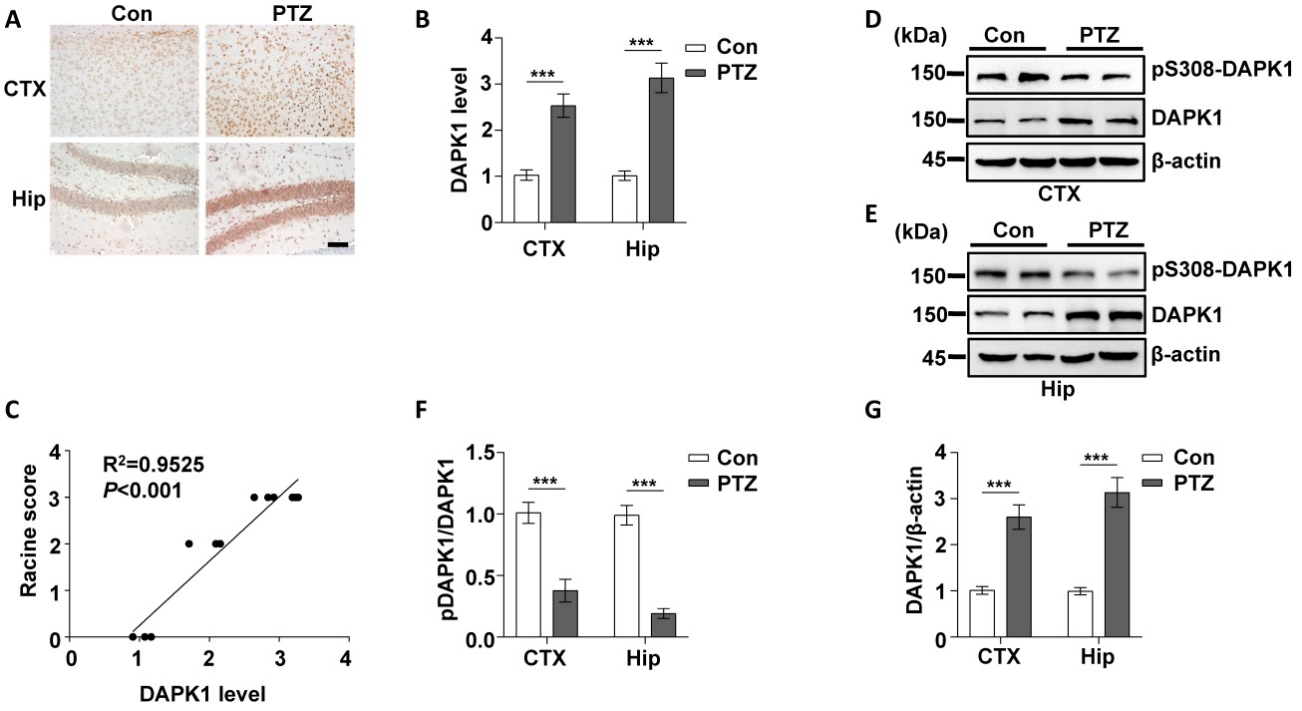
** Kindling by repeated PTZ administration promotes DAPK1 protein expression in mice.** Mice (C57BL/6, male, P56-P80, n = 8/group) were treated with a subconvulsive dose of PTZ (35 mg/kg) every other day (four injections for 7 days), and brain tissues were harvested 30 min after the fourth PTZ injection. **A** Immunohistochemistry using an anti-DAPK1 antibody was conducted on paraffin-embedded cortical and hippocampal sections from the control and PTZ-treated mice. Scale Bar = 100 µm. **B** Quantitation of DAPK1 staining intensity (arbitrary units; two-tailed Student's t test). **C** Correlation between the Racine score on the Y axis and DAPK1 expression on the X axis (R^2^ = 0.9525,* P* < 0.01, Pearson correlation coefficient). **D-G** Cortical and hippocampal lysates were analyzed via immunoblotting with an anti-pSer308-DAPK1, anti-DAPK1 or anti-β-actin antibodies (^***^P < 0.001 *vs* control; two-tailed Student's t tests). All data shown represent the means ± standard errors of three independent experiments.

Epilepsy is generally characterized by spontaneous recurrent seizures (SRSs) 52. However, a single administration of PTZ does not induce chronic spontaneous and recurrent seizures; thus, repeated administration of PTZ is required to induce SRS 53. The PTZ kindling model has been widely used to investigate the pathophysiology of epilepsy 54. Mice are generally considered fully kindled after exhibiting three consecutive convulsive seizures. Since DAPK1 is activated by a single dose of PTZ, we investigated whether kindling by repeated PTZ administration affects DAPK1 expression by immunohistochemical staining of a series of brain sections using a specific antibody against DAPK1. As shown by immunohistochemistry, the expression of DAPK1 was significantly elevated in the cortical and hippocampal regions of repeated PTZ-treated mice compared with age-matched control mice (Fig. 4A and B). Immunoblotting assays also confirmed that DAPK1 was highly overexpressed in the cortex and hippocampus after repeated PTZ administration (Fig. 4D, E and G). Moreover, phosphorylated Ser308 DAPK1 levels were decreased in both the cortex and hippocampus (Fig. 4D-F), indicating that repeated PTZ administration increases both DAPK1 expression and activity. During the progression of epilepsy in the PTZ-kindling model of chronic epilepsy, the seizure grade was increased, which was positively correlated with DAPK1 expression levels as evidenced by the Pearson correlation coefficient (R^2^ = 0.9525) (Fig. 4C). Therefore, the data demonstrated that DAPK1 levels and activity are increased in the PTZ-kindled chronic epilepsy mouse model and that this level might be positively correlated with the seizure grade.

### Suppression of DAPK1 expression or activity reduces the development of kindled seizures

**Figure 5:**
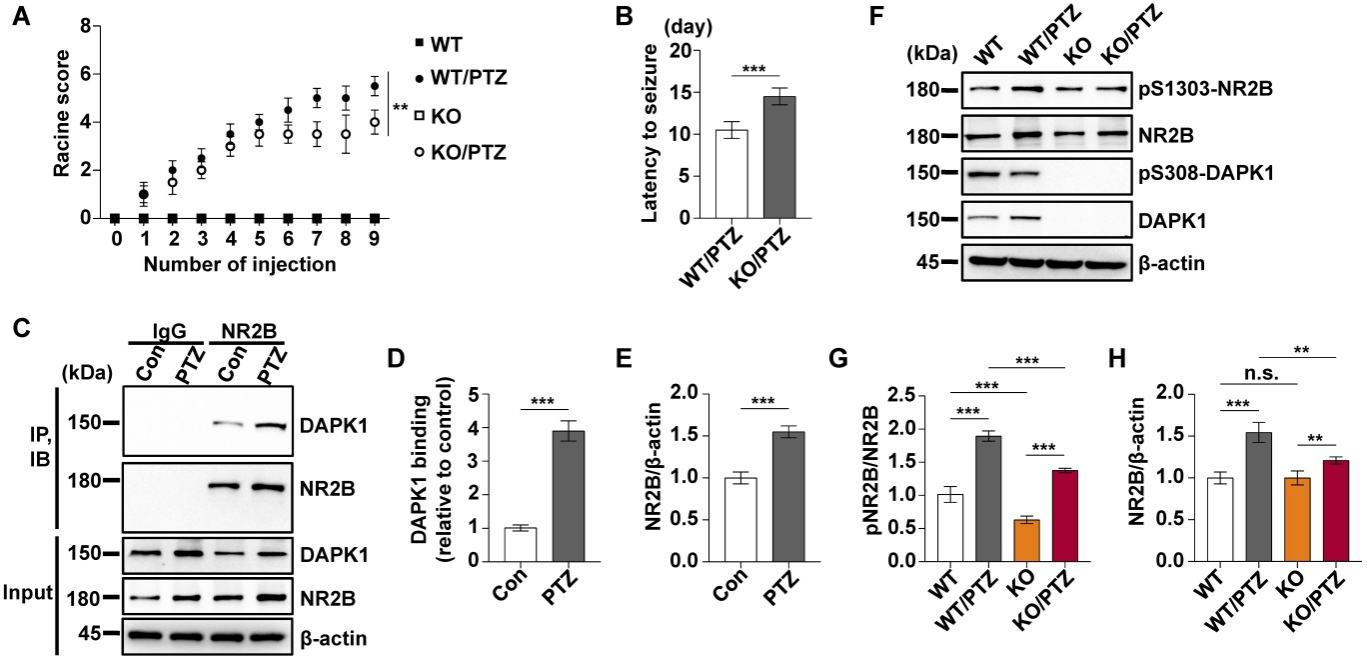
** DAPK1-deficient mice display a significant delay in the development of kindling.** Mice (C57BL/6, male, P56-P80, n = 12/group) were treated with a subconvulsive dose of PTZ (35 mg/kg) every other day (nine injections for 17 days). The brain tissues from a number of the animals (n = 4) were harvested after the fourth PTZ injection, and the remaining animals continued to undergo behavioral analysis. **A** The mean seizure grades of PTZ-kindled WT and DAPK1 KO mice; DAPK1 KO mice exhibited significantly prolonged latency to the first kindled seizure scored ≥ 4 (^**^P < 0.01, one-way ANOVA with Tukey's multiple comparisons test). **B** Latency to the first kindled seizure was significantly prolonged in the DAPK1-deficient mouse group (^***^P < 0.001, two-tailed Student's t test). **C-E** Enhanced interaction between NR2B and DAPK1 in the hippocampus after PTZ exposure (^***^P < 0.001 *vs* control, two-tailed Student's t tests). **F-H** Brain lysates were analyzed via immunoblotting with anti-pSer1303-NR2B, anti-NR2B, anti-pSer308-DAPK1, anti-DAPK1, or anti-β-actin antibodies. Statistical significance was determined by one-way ANOVA with Tukey's multiple comparisons tests (^**^P < 0.01 and ^***^P < 0.001). n.s., no significance. All data shown represent the means ± standard errors of three independent experiments.

**Figure 6:**
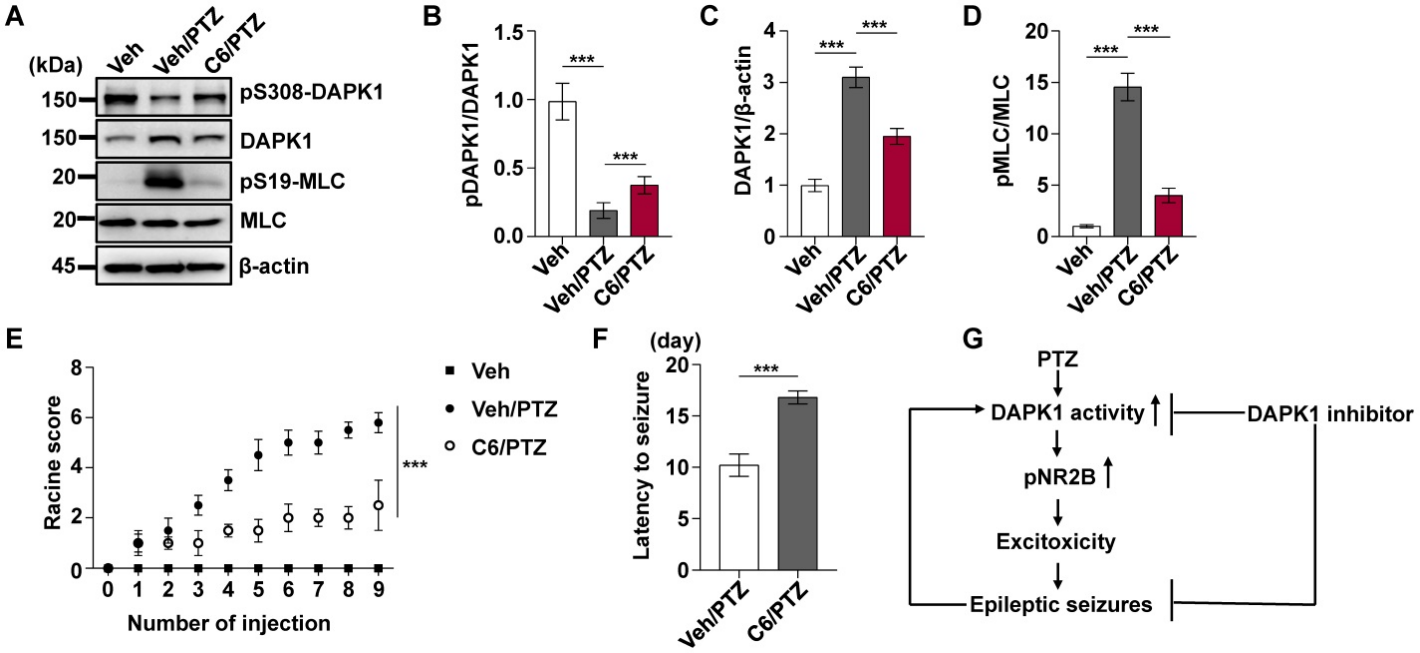
** Pharmacological inhibition of DAPK1 reduces the development of epilepsy in PTZ-induced kindling.** Mice (C57BL/6, male, P56-P80, n = 12/group) were pretreated with a DAPK1 inhibitor (10 mg/kg) or vehicle for 5 min followed by a subconvulsive dose of PTZ (35 mg/kg) every other day (nine injections for 17 days). The brain tissues from a number of the animals (n = 4) were harvested after the fourth PTZ injection, and the remaining animals continued to undergo behavioral analysis. **A-D** DAPK1 inhibitor pretreatment decreased DAPK1 activity after chronic PTZ exposure. The brain lysates were subjected to immunoblotting analysis with anti-pSer308-DAPK1, anti-DAPK1, anti-pSer19-MLC, anti-MLC or anti-β-actin antibodies. Statistical significance was determined by one-way ANOVA with Tukey's multiple comparisons tests (^***^P < 0.001). **E** Mean seizure grades of PTZ-kindled WT treated with vehicle or C6, with lower seizure grades were noted in the DAPK1 inhibitor-treated mouse group (^***^P < 0.001, one-way ANOVA with Tukey's multiple comparisons test). **F** Latency to the first kindled seizure was significantly prolonged in the DAPK1 inhibitor pretreatment mouse group (^***^P < 0.001* vs* vehicle, two-tailed Student's t test). All data shown represent the means ± standard errors of three independent experiments. **G** Schematic diagram summarizing the proposed role of DAPK1 in epilepsy.

To investigate the effect of DAPK1 inhibition on the development of kindled seizures, we first examined whether the suppression of DAPK1 expression plays a protective role in epilepsy by administrating repeated PTZ doses of 35 mg/kg in the WT and DAPK1 KO mice. During the course of the PTZ kindling experiment, mice displayed a progressive increase in seizure scores, beginning with almost undetected convulsive behaviors and eventually progressing to the first kindled seizure. PTZ-treated DAPK1 KO mice displayed a significant delay in the development of kindling under current conditions (latency to the first kindled seizure of 10.5 days *vs* 14.5 days for the WT and DAPK1 KO groups, respectively) (Fig. 5A and B). While the total NR2B levels were not changed by a single dose of PTZ (Fig. 2H), NR2B expression was moderately increased after repeated PTZ injections (Fig. 5F and H). Moreover, DAPK1 was more strongly associated with NR2B in the hippocampus after repeated PTZ treatment compared with the control mice (Fig. 5C-E). Compared with the saline-treated WT mice, phosphorylated NR2B levels were highly elevated in the hippocampal regions of the WT mice subjected to repeated PTZ administration (Fig. 5F-H). However, the phosphorylation of NR2B at Ser1303 was decreased after PTZ kindling in DAPK1 KO mice (Fig. 5F-H). We next examined the effect of the DAPK1 inhibitor on PTZ-induced kindled seizures. Mice were treated with a subconvulsive dose of PTZ (35 mg/kg) every other day, and one group of animals was pretreated with a DAPK1 inhibitor (10 mg/kg) for 5 min before each PTZ treatment. Brain tissues from a number of the animals were harvested after the fourth PTZ injection, and the remaining animals continued to be evaluated for a behavioral analysis until the end of the experiment. Compared with the untreated mice, the DAPK1 inhibitor increased the levels of phosphorylated DAPK1 at Ser308 and decreased phosphorylated MLC levels with PTZ administration (Fig. 6A, B and D). While DAPK1 expression was markedly increased after repeated PTZ administration, DAPK1 levels were moderately increased in the DAPK1 inhibitor-pretreated mouse group after repeated PTZ treatment (Fig. 6A and C). The increases in seizure grade by PTZ stimulation were significantly retarded for 9 stimulations (seizure stage of 5.8 *vs* 2.5 for vehicle-treated group *vs* C6-treated group, respectively), and more than 90% of the DAPK1 inhibitor-treated mice could not develop the first kindled seizure (latency to the first kindled seizure of 10.2 days *vs* 16.8 days for the vehicle-treated group and C6-treated group, respectively; *P* < 0.001) (Fig. 6E and F). Thus, these results indicate that suppressing the expression or activity of DAPK1 through specific inhibitors significantly reduces the development of kindled seizures by affecting NMDAR signaling.

## Discussion

Although previous studies indicate that DAPK1 may be important for the development of epilepsy, the molecular mechanisms of DAPK1 and its therapeutic efficacy against epileptic seizures remain largely unresolved. In this research, we proved that DAPK1 is regulated by dual mechanisms depending on the seizure conditions and showed that the inhibition of DAPK1 expression or activity has an antiepileptic effect. Epilepsy is a very complex disease that progresses rapidly. According to the EEG and behavioral characteristics, a convulsive seizure induced by PTZ consists of at least three stages, including GTCS, PGES, and post-PGES 37. We found that DAPK1 may be regulated by its activity in the early stage and then by its activity/expression in the later stage. We also found that the level of phosphorylation of DAPK1 at Ser308 was dramatically decreased upon acute PTZ treatment. Reports have indicated that DAPK1 activity is negatively regulated in a Ca^2+^/calmodulin-binding autoregulatory domain at Ser308 by autophosphorylation 19, 23, 24. Its phosphorylation inhibits calmodulin binding to the catalytic cleft in the domain and suppresses DAPK1 catalytic activity. Although several phosphatases have been identified as dephosphorylated at Ser308, including protein phosphatase 2A, further studies are required to identify which phosphatases regulate DAPK1 activity by dephosphorylating Ser308 in PTZ-induced epileptic stresses.

Interestingly, we discovered that the expression and activity of DAPK1 was upregulated in the epilepsy model induced by chronic PTZ administration. Immunohistochemistry and immunoblotting assays showed that DAPK1 levels were upregulated in both the cortex and hippocampus and positively correlated with the level of seizure grade. In the acute PTZ model, PTZ-induced epilepsy phenotypes occur within minutes, including tonic-clonic seizure 55. Seizures are accompanied by a significant increase in excitatory amino acids in the brain, including glutamate 56. The binding of glutamate to glutamate receptors in the brain mediates a large influx of calcium ions, leading to the increased DAPK1 activity 22. Activated DAPK1 phosphorylates p53 at Ser23 and promotes p53 nuclear localization, thereby increasing its transcriptional ability 57. Since DAPK1 is a transcription target of p53 58, DAPK1 expression might be increased by a positive feedback manner in the PTZ kindled model. Under current experimental setting by collecting brain samples after acute PTZ treatment within 3 min, we did not detect the increased levels of DAPK1 expression. Thus, DAPK1 activity might be induced by the calcium influx in the acute PTZ treatment and DAPK1 expression might be increased by p53 activation via activated DAPK1 in the kindled model. Recently, we discovered that melatonin enhances DAPK1 protein degradation via the ubiquitin-mediated proteasome pathway 51, 59. Since melatonin has been shown to suppress epilepsy-related seizures and DAPK1 has been targeted by E3 ubiquitin ligases 23, 60, melatonin may regulate DAPK1 levels in a posttranslational manner in the development of kindled seizures.

Reports have indicated that the enhanced interaction between DAPK1 and NR2B acts as an important mediator of irreversible neuronal damage in stroke through increased NMDAR channel conductance and subsequent injurious calcium influx 22. Uncoupling DAPK1 from NR2B protects neurons against cerebral ischemic attack by blocking Ca^2+^ influx through the NR2B channel 22. High expression of DAPK1 and phosphorylated NR2B at Ser1303 are key components in the pathophysiology of depression 61. In our study, DAPK1 activity was increased but its expression was not by the acute PTZ treatment, which mediates NR2B phosphorylation at Ser1303 and accelerates the formation of GTCS. Moreover, we found that overexpressed DAPK1 directly binds to NR2B and increases phosphorylated NR2B levels, thereby increasing seizure severity induced by long-term PTZ exposure in the kindling model. These results indicated that both activated and overexpressed DAPK1 enhance NR2B binding and phosphorylation under seizure conditions. Furthermore, DAPK1 interacts with TNFR1 or p53 and increases neuronal apoptosis in seizures 32. Recently, overexpressed DAPK1 in endothelial and astrocytic cells from epilepsy patient brains was shown to regulate cell death by hypoxia in epileptic pathological conditions 30. Future studies should include intensive cross-talk between DAPK1 and its interacting partners and/or downstream targets in the development of epilepsy.

To further corroborate our findings, we used DAPK1 KO mice to investigate the effects of DAPK1 ablation on PTZ-induced seizures. We found that the inhibition of DAPK1 expression showed lower seizure scores, longer latency and lower amplitude of GTCS on acute PTZ administration and displayed a significant delay in the development of kindling compared to WT littermates. Mechanistically, NR2B phosphorylation was attenuated in DAPK1 KO mice, suggesting that DAPK1 ablation decreased seizure susceptibility *in vivo* and has therapeutic potential for epilepsy. In addition, we demonstrated that selective inhibition of DAPK1 by specific inhibitors can be a promising approach to suppressing epileptic seizures. Based on the finding that pharmacological inhibition of DAPK1 activity produces rapid and potent antiseizure effects in both PTZ-induced acute and kindling animal models, DAPK1 inhibition might be a promising option for epilepsy interventions. However, special consideration is needed to overcome the potential side effects of DAPK1 inhibition by modulating DAPK1 activity because DAPK1 also plays a part in regulating cell growth and tumor suppression 23. More studies are required to clarify whether the DAPK1 inhibitor could penetrate the blood-brain barrier to suppress DAPK1 activity and/or exert antiepileptic effects in a tissue-specific manner. Furthermore, it should be warranted whether the combination treatment of melatonin and DAPK1 inhibitor has synergistic effects on epilepsy. Such studies may offer valuable insights for developing disease-modifying treatments for epilepsy.

In summary, our data were used to develop a model in which PTZ promotes DAPK1 activity through or increases DAPK1 expression and directly phosphorylates NR2B, which results in increased excitotoxicity and epileptic seizures (Fig. 6G). Ablation of DAPK1 expression by gene KO or suppression of DAPK1 activity by a small molecule significantly reduces seizure phenotypes and epilepsy development. Our study has thus deciphered a key role of DAPK1 in modulating PTZ-induced seizures and could provide insights on the development of novel therapeutic approaches for human epilepsy.

## Supplementary Material

Supplementary figure and table.

## Abbreviations

DAPK1: Death-associated protein kinase 1
PTZ: pentylenetetrazol
EEG: Epidural electroencephalogram
GTCS: generalized tonic-clonic seizure
PGES: postictal generalized EEG suppression
NMDAR: N-methyl-D aspartate receptor
