# Supplementary material for: Inhibition of Death-associated Protein Kinase 1 protects against Epileptic Seizures in mice
Source: Int J Biol Sci. 2021 Jun 11;17(9):2356–66. doi: 10.7150/ijbs.59922 (PMC8241737; doi:10.7150/ijbs.59922)
Supplement: Supplementary file 1 — Supplementary figure and table. [file ijbsv17p2356s1.pdf]

Supplementary Materials

Gan et al.

Inhibition of Death-associated Protein Kinase 1 Protects against Epileptic Seizures in Mice

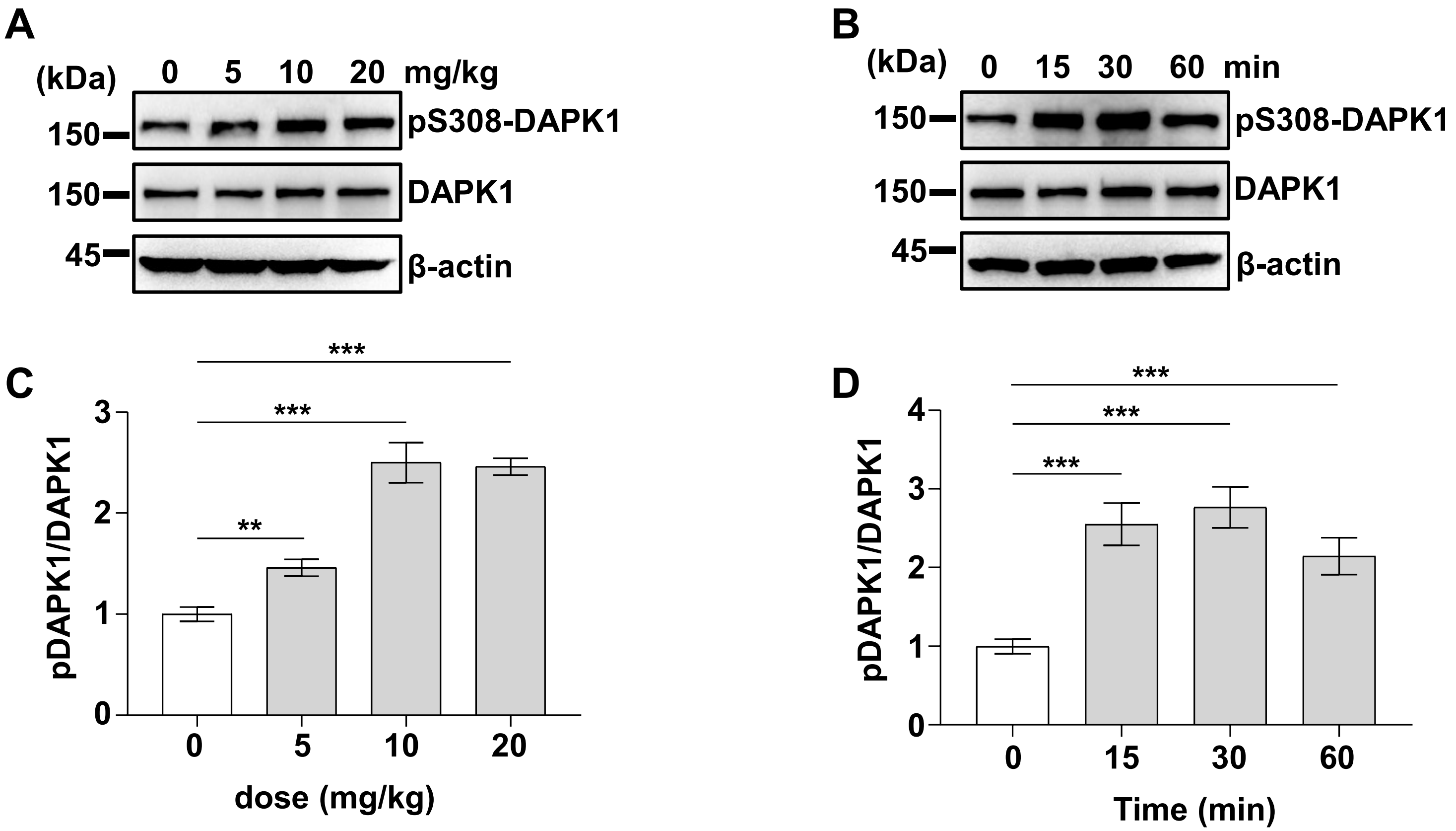

Fig. S1 DAPK1 inhibitor (C6) suppresses DAPK1 activity in mouse brain.
A Mice (C57BL/6, male, P56-P80, n = 5/group) were administered different dose of C6, and brain tissues were harvested 30 min after C6 injection. **B** Mice (C57BL/6, male, P56-P80, n = 5/group) were administered C6 (10 mg/kg), and brain tissues were harvested at different time point after C6 injection. Hippocampal lysates were subjected to immunoblotting analysis with anti-pSer308-DAPK1, anti-DAPK1, and anti-β-actin antibodies. **C-D** Statistical significance was determined by one-way ANOVA with Tukey's multiple comparisons tests (**$P < 0.01$, ***$P < 0.001$). All data shown represent the means ± standard errors of three independent experiments.

Supplementary Table 1 Primary antibodies used in this study.

Antibody	Dilution	Source	Identifier
Mouse anti-DAPK1	1:1000 (WB)	MilliporeSigma	D2178
Rabbit anti-DAPK1	1:50 (IHC)	MilliporeSigma	SAB4500620
Mouse anti-pS308 DAPK1	1:1000 (WB)	Abmart	
Mouse anti-pS308 DAPK1	1:1000 (IHC)	MilliporeSigma	D4941
Mouse anti-MLC	1:1000 (WB)	Abcam	ab233152
Mouse anti-pMLC (Ser19)	1:1000 (WB)	Cell Signaling Technology	3675
Rabbit anti-NR2B	1:1000 (WB)	Abcam	65783
Rabbit anti-pNR2B (Ser1303)	1:1000 (WB)	MilliporeSigma	07-398
Mouse anti-β-actin	1:20000 (WB)	MilliporeSigma	A5441

WB, Western blotting; IHC, Immunohistochemistry
